# 1656. Evaluation of Using Oxacillin versus Vancomycin for Empiric Therapy of Late-Onset Neonatal Sepsis in NICU

**DOI:** 10.1093/ofid/ofad500.1489

**Published:** 2023-11-27

**Authors:** Tsung-Chi Lien, Harlan Husted, Sahar Taha, Alice Ip, Laurie Covarrubias, Kathleen Henschel, Maria Concepcion Mendoza, Anand Rajani, Chokechai Rongkavilit

**Affiliations:** Community Regional Medical Center, Fresno, CA; Community Regional Medical Center, Fresno, CA; Community Regional Medical Center, Fresno, CA; Community Regional Medical Center, Fresno, CA; Community Regional Medical Center, Fresno, CA; Community Regional Medical Center, Fresno, CA; University of California San Francisco, Fresno Branch Campus, Fresno, California; Community Regional Medical Center; University of California San Francisco, Fresno Branch Campus, Fresno, California; University of California San Francisco, Fresno Branch Campus, Fresno, California

## Abstract

**Background:**

Our Pediatric Antimicrobial Stewardship Program (PedASP) assessed methicillin-sensitive (MSSA) and methicillin-resistant *S. aureus* (MRSA) infections in neonatal intensive care unit (NICU) and created a NICU-specific antibiogram. Due to a rising trend of NICU MSSA infections seen in our 84-bed NICU, empiric therapy of late-onset neonatal sepsis (LONS) was modified from vancomycin to oxacillin in January 2021. We report the two-year results of using empiric oxacillin for LONS.

**Figure 1.**
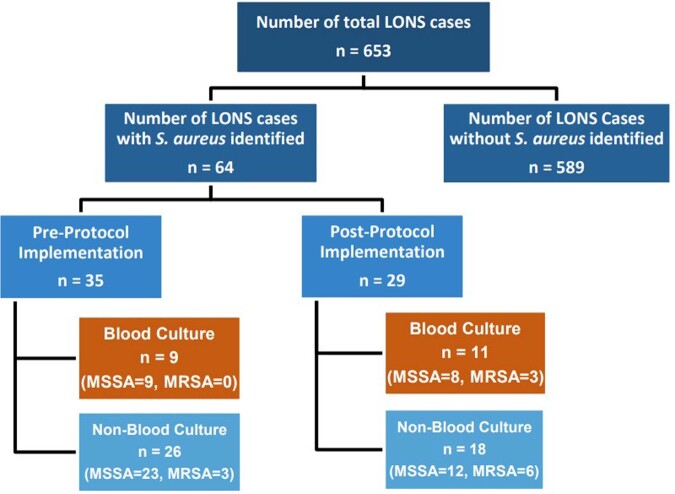
Summary of S. aureus identified in LONS cases

**Methods:**

We assessed MSSA versus MRSA isolated from blood and non-blood cultures in NICU between January 2019 and December 2022. Infants with weight less than 750 grams, previous MRSA infections, maternal MRSA colonization, or serious infections based on clinical judgement were advised to use empiric vancomycin. We examined the vancomycin eligibility criteria, antibiotic-pathogen mismatch rate, 30-day mortality, and frequency of peripheral intravenous (PIV) access loss after vancomycin or oxacillin administration. The cost-savings was estimated by comparing drug and lab costs associated with vancomycin and oxacillin use.

**Results:**

LONS cases are summarized in Figure 1. There was no statistical difference in incidence of *S. aureus* bacteremia before and after protocol implementation (p=0.45). Antibiotic-pathogen mismatch rate was 0%. Cases of MRSA bacteremia increased from 0 to 3 post protocol initiation; all cases met vancomycin eligibility criteria. The 30-day mortality among MRSA infections increased from 0 to 1 case. Post protocol implementation, the PIV loss rate decreased from 4.7 to 3.3% (p=0.04), and vancomycin use decreased by 1154 doses while oxacillin use increased by 485 doses. Based on antibiotic use and 141 vancomycin levels averted, the estimated cost-savings was $140,229.

**Conclusion:**

Our PedASP utilized local susceptibility data to change empiric antibiotics for LONS, reinforcing the benefit of using antibiogram to optimize antibiotic use in NICU. Implementing a NICU-specific antibiogram improved the effectiveness of antibiotic treatment and achieved significant cost savings.

**Disclosures:**

**All Authors**: No reported disclosures

